# Neuromuscular Adaptations and Enhancement of Physical Performance in Female Basketball Players After 8 Weeks of Plyometric Training

**DOI:** 10.3389/fphys.2020.588787

**Published:** 2021-01-21

**Authors:** Yosser Cherni, Mehrez Hammami, Mohamed Chedly Jelid, Ghaith Aloui, Katsuhiko Suzuki, Roy J. Shephard, Mohamed Souhaiel Chelly

**Affiliations:** ^1^Research Unit (UR17JS01) “Sport Performance, Health & Society”, Higher Institute of Sport and Physical Education of Ksar Saîd, University of “La Manouba”, Tunis, Tunisia; ^2^Higher Institute of Sport and Physical Education of Ksar Said, University of “La Manouba”, Tunis, Tunisia; ^3^Faculty of Sport Sciences, Waseda University, Tokorozawa, Japan; ^4^Faculty of Kinesiology and Physical Education, University of Toronto, Toronto, ON, Canada

**Keywords:** ability to change direction, vertical jumping, electromyographic activity, peak torque, peak power

## Abstract

The aim of this study was to examine the effects of an 8-week in-season plyometric training (PT) program on the physical performance and neuromuscular adaptations of female basketball players. Twenty-seven elite female basketball players (aged 21.0 ± 2.6 years) were assigned between an experimental group (*n* = 15) who substituted a part of their usual training with biweekly PT, and a control group (*n* = 12) who maintained their standard basketball training. Analyses of variance and co-variance assessed changes in 10, 20, and 30 m sprint times, ability to change direction (*T*-test) and jumping ability [squat jump (SJ) and countermovement jump (CMJ)] with electromyographic assessment of the vastus lateralis, vastus medialis, and rectus femoris muscles during jumping and meassurement of the isokinetic strength of the knee muscles. After 8 weeks of the plyometric program the experimental group enhanced change of direction performance (Δ = −3.90%, *d* = 0.67) and showed a greater thigh cross sectional area (Δ = 9.89%, *d* = 0.95) relative to controls. Neural adaptations included significant improvements of EMG parameters for the vastus medialis muscle during Squat Jumping (Δ = 109.3%, *d* = 0.59). However, trends to improvements of sprinting times and jumping performances did not reach statistical significance. In addition, there were no gains in the peak torque and the average power of the quadriceps and hamstring muscles at either slow or moderate test speeds. We conclude that 8-weeks of PT (72–126 jumps) was insufficient to improve many of the variables associated with basketball performance in our subject-group. Further studies of female basketball players, extending the program period and increasing the intensity and speed of jumps are recommended in the search for more significant results.

## Introduction

Among potential conditioning techniques, plyometric training (PT) is recognized as a safe and effective method of improving explosive actions. Plyometric exercises involve repeated stretch-shortening cycles (SSC), a rapid muscle stretch (eccentric phase) being followed immediately by a rapid shortening of the muscle (concentric phase) (Markovic and Mikulic, 2010). This pattern of contraction has been shown to enhance sprinting ability (Sáez de Villarreal et al., 2012), agility (Miller et al., 2006), muscle power and strength (Saez-Saez de Villarreal et al., 2010), and jump height (Markovic et al., 2007).

The ability to reach maximal strength in the shortest period of time is crucial to the performance of several sports, particularly basketball (Santos and Janeira, 2008). Basketball is an intermittent sport, characterized by frequent and sudden variations in the intensity of movement in different planes (Ben Abdelkrim et al., 2007). Players perform 18–105 sprints during 2–6% of total game time (Ben Abdelkrim et al., 2007; Scanlan et al., 2011) and 26.0 ± 7.6 changes of direction (Conte et al., 2015) can occur at intervals of 2–3 s (Delextrat and Cohen, 2009). Jumping is also essential during rebounding, shooting or blocking, with means of 35–46 jumps per game in male players (McInnes et al., 1995; Matthew and Delextrat, 2009) and 19–43 jumps in female players (Ziv and Lidor, 2010). During competition, players have to overtake their opponents by being faster and more powerful. Hence, an improvement of speed and acceleration as well as an increases in strength and power are crucial to maximizing basketball performance.

Plyometric training has become an important component of fitness programs for several team sports such as soccer (Bianchi et al., 2019), handball (Chelly et al., 2014), volley ball (Silva et al., 2019), and basketball (Cherni et al., 2019). However, there is as yet limited information from controlled trials of PT in adult female basketball players (Ramirez-Campillo et al., 2018a), particularly over training periods of more than 7 weeks (Stojanovic et al., 2017; Ramirez-Campillo et al., 2018b). More information is needed on potential gains in sprinting (Sáez de Villarreal et al., 2012), jumping (Stojanovic et al., 2017), change of direction ability (Asadi et al., 2016), and the mechanisms underlying any enhancements of muscular power (Saez-Saez de Villarreal et al., 2010) as well as the effects of PT on body composition and anatomic adaptations (Saez-Saez de Villarreal et al., 2010) such as changes in the percentage fat, leg muscle volume and mean cross sectional area, particularly in female athletes. Moreover, the plyometric programs adopted in previous studies of adult female players were derived from studies on male players; however, there are sex differences with regard to muscle composition (with dominance of type I muscle fibers in women) and muscle characteristics (fascicle length and pennation angles) and in the ability to use SSC (which is 64.1% in women that of men (Saez-Saez de Villarreal et al., 2010). Hence, the optimal jumping training volume and intensity stimulus for adult female players remains to be resolved.

We thus investigated the effects of 8 weeks of biweekly PT on sprinting speeds, change of direction ability, vertical jumping ability [squat (SJ) and counter movement (CMJ) jumps], electromyographic (EMG) activity] and the peak torque and average power of both legs in this category of team athletes. Our hypothesis was that the substitution of a part of a standard in-season skills-based training regimen by an 8-week PT program would enhance each of these measures of playing ability.

## Materials and Methods

### Participants

The G power 3.0.10 program was used to calculate the minimal sample size needed in our study, with Z1-β = 1.03 (power = 85%) and *Z*_/2_ = 1.96 (α = 5%). The study of Meszler and Váczi (2019) showed the mean ± SD of counter movement vertical jump as 33.52 ± 3.89 (cm) in the experimental group vs. 28.72 ± 6.66 (cm) in the control group, and considering a ratio of 1 control for every case, there was thus a need for a minimum of 11 experimental and 11 control subjects (Faul et al., 2007).

All participants were first informed about the planned training and testing, together with associated benefits and risks. They were assured that they could withdraw from the trial without penalty at any time. Twenty-seven healthy elite female basketball players (aged 21.0 ± 2.6 years), who had been participating in competitions for at least 5 years volunteered to serve as subjects. Participants had to be free from any illness or disease that could affect their performance and injury free during the preceding 6 months. They were recruited from two First Division National League teams with approximately the same ranking and were assigned between an experimental group (*n* = 15) and a control group (*n* = 12). Participants’ characteristics are detailed in Table 1. All were examined by the team physician, with a particular focus on orthopedic or other conditions that might proscribe involvement in the program, and all were found to be in good health. From the beginning of the season, each player was questioned whether she had a regular menstrual cycle, the length of her cycle and if she was using any hormonal contraception. All players had a regular cycle (24–32 days) and none of them were taking contraceptives. During the tests (pre and post) the phases of each players’ cycles were determined by using a menstrual calendar.

**TABLE 1:** Initial characteristics of experimental and control groups.

	Experimental group (*n* = 15)	Control group (*n* = 12)
Age (years)	20.9 ± 2.4	21.0 ± 3
Height (m)	1.72 ± 0.06	1.73 ± 7.24
Body mass (kg)	65.1 ± 8.8	67.3 ± 10.6
BMI (kg/m^2^)	21.9 ± 2.0	22.5 ± 2.7
Years of basketball practice	10.8 ± 3.2	10.8 ± 4.8

*n, number. Data are presented as means ± SD.*

Participants agreed not to change their personal exercise habits during the course of the intervention. The annual training schedule of the subjects is detailed in the Table 2.

**TABLE 2:** Annual training program and competition for all players in the current investigation.

Period		Number of weeks	Number of sessions/week	Program design					
***Preparatory***	***Initial phase***	4 weeks (September)	4	- Capacity workout: technical basketball exercises.					
***period***				- Aerobic capacity training for 2 first weeks and power capacity training for 2 next weeks.					
			2	- Light resistance training: (40–60% 1 repetition maximum [RM]).					
	***Second phase***	4 weeks (October)	2	- Light resistance training: higher loads were used (70–85% 1RM).					
			4	- Intermittent all-court basketball drills with offensive superiority challenges and					
				participation in 1 friendly game each weekend.					
				- Maximal power capacity training for all weeks of second part.					

***First phase of competition***		12 weeks (November–January)	4–5 + 1 official game day	**Mondays**	**Tuesdays**	**Wednesdays**	**Thursdays**	**Fridays**	**Saturdays**

***Rest period***		2 weeks (beginning February) **Allocation for the testing protocol and familiarization**		An integrated aerobic training based on continuous play	An integrated strength power training characterized by defensive and circuit training	An integrated maximum power aerobic training made up of technical drills at various intensities	An integrated training based on counter-attacks and sprints and agility training	A liveliness involving offensive and defensive tactics and dexterity and vivacity training	Official games
***Second phase of competition***		10 weeks (2nd half of February-April) **Current plyometric program**							
***Final phase of competition***		4 weeks May							

### Procedures

The study examined the impact of an 8-week in-season PT program (two sessions per week, with a 48-h rest interval) on sprinting, jumping, agility, peak power, average power and associated neuromuscular adaptations in elite female basketball players, comparing findings post-intervention to data for their peers who had continued to follow the standard technical and tactical in-season training program. The intervention was performed mid-season, during an 8-week period from February to April.

All procedures were conducted in accordance with the latest version of the Declaration of Helsinki, and were approved by the University of Manouba [Research Unit (UR17JS01) «Sport Performance, Health & Society», Higher Institute of Sport and Physical Education of Ksar Saîd, University of “La Manouba”, Tunis, Tunisia] Institutional Review Committee for the ethical use of human subjects, according to current national laws, regulations and procedures.

### Testing Schedule

Two weeks before the initial experimental measurements, participants were familiarized with all test protocols. Formal testing was incorporated into the weekly training schedule, 48 h after the game day, with data collected prior to and after completion of the plyometric program. Initial and final testing sessions were conducted at the same time of day (09:00–12:00 am) and under approximately the same environmental conditions (20–25°C); the experimental group undertook their final testing at least 5 days after their last PT session. Participants were fully hydrated, but abstained from caffeine-containing beverages for 4 h, and food for 2 h before testing. They also had a full night’s sleep prior to assessment. Standardized warm-up exercises preceded testing, which was conducted on a parquet floor, following a consistent test order over the course of 3 days. The protocol included assessments of sprinting ability (10, 20, and 30-m sprint times), a change of direction test (*T*-test) counter-movement jumps tests, with EMG recordings from the vastus lateralis (Markovic and Mikulic, 2010), vastus medialis (VM), and rectus femoris (RF) muscles during jumping. Idsokinetic measurements of leg muscle strength were made during the last test day.

#### Anthropometrics

The body height and mass were assessed using a stadiometer and weighing scales, respectively. Skin folds were measured using a standard Harpenden caliper (Baty International, Burgess Hill, Sussex, United Kingdom). The overall percentage of body fat was estimated from the biceps, triceps, subscapular, and suprailiac skinfolds, using the formulae (Durnin and Womersley, 1974):

%Bodyfat=(4.95/[Density-4.5])100

where density = 1,1549 – 0,0717 (log_10_ (sum of 4 skinfolds)) and 1,1549 and 0,0717 are sex and age dependent constants.

#### Leg Muscle Volume

Circumferences and skin-fold thickness at different levels of the thigh and the calf, the length of the leg and the width of the condyles of the knee were measured to estimate the leg muscle volume, as described previously (Jones and Pearson, 1969; Shephard et al., 1988). The accuracy of this anthropometrical method was validated by comparison with the dual-energy x-ray absorptiometry (DEXA) method (Chelly et al., 2006). The leg muscle volume was estimated as follows (Jones and Pearson, 1969):

Musclevolume=totallimbvolume-(fatvolume+bonevolume)

The total limb volume was estimated as the volume of a cylinder determined by:

the height (H) of a cylinder corresponding to the distance from the trochanter major to the external malleolus of the ankle and a basal area corresponding to the mean area of five circumferences around the limb (maximal of the thigh, mid-thigh, just below the patella, maximal of the calf and just above the ankle)

Thetotallimbvolumewascalculatedas:((∑C2)×H/62.8)

where ∑C2 is the sum of the squares of the five circumferences (Jones and Pearson, 1969).

• Fat volume = (∑C/5) × (∑S/2n) H (Jones and Pearson, 1969)

where ∑S is the sum of four skinfolds (front of midthigh, back of midthigh, back of the calf and outside of the calf) as determined with a standard Harpenden caliper (Baty International, Burgess Hill, Sussex, United Kingdom) and n corresponds to the number of folds.

• Bone volume = p × (F × D)^2^ × L (Jones and Pearson, 1969)

where *D* is the femoral intercondylar diameter and *F* is a geometrical factor (equal to 0.235 for the leg, i.e., average bone radius = 23.5% of the femoral intercondylar diameter).

#### Mean Cross Sectional Area (CSA) of the Thigh (Muscle Plus Bone)

The mean thigh CSA was calculated from maximal and mid-thigh circumferences.

Considering the circumference of the thigh as a circle, the surface of the transversal section, after allowance for the overlying skin folds (the sum of anterior and posterior skinfolds) is calculated as (Chelly et al., 2006):

$$\begin{array}{c} \mathrm{Circumference}\left(C\right)=2\pi \cdot \mathrm{Radius}\left(R\right)\\ R=C/2\pi \end{array}$$

r is the radius of the transversal section of the mid-thigh.

r = R – [(mid-thigh anterior skin fold + mid-thigh posterior skin fold)/4] (Chelly et al., 2006)

The surface of the transversal section of the thigh at each level, CSA, equaled π r^2^ (cm^2^).

#### Sprint Performance

Sprint tests were conducted in a gymnasium, on a parquet floor. Sprint times over distances of 10, 20, and 30 m were recorded using four single-beam photoelectric gates (Microgate, Bolzano, Italy), mounted at a height of 0.75 m. Players began bending slightly forward, with the dominant leg at 0.2 m from the first photocell beam. Three trials were separated by 5-min recovery intervals, with the fastest results being noted.

#### Ability to Change Direction (*T*-test)

**FIGURE 1:**
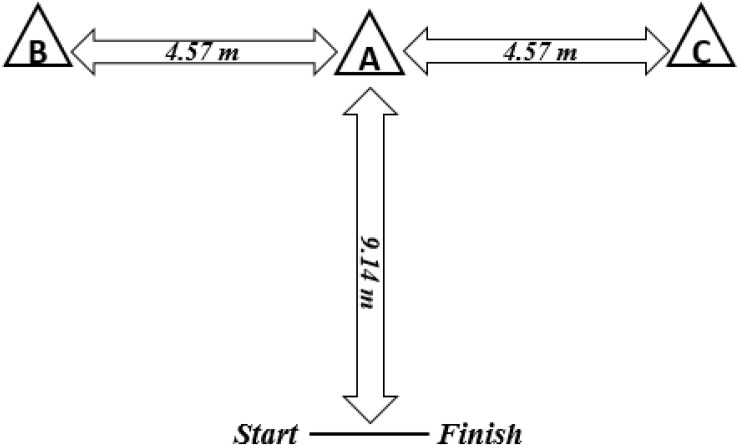
*T*-test schema.

Times of movements of forward running from the start line up to cone A, followed by lateral shuffles to the left (cone B), then to the right (cone C), then go back to cone A and finally running back to the finish line were reported (Pauole et al., 2000), using an electronic timing gate (Microgate SARL, Bolzano, Italy) (Figure 1). The test was repeated three times with 5-min rest intervals, and the fastest times were recorded.

#### Squat and Counter-Movement Jumps

Squat- and counter-movement jumps heights were assessed using a force platform (Quattro Jump, version 1.04; Kistler Instrument AG, Winterthur, Switzerland). During the squat jump, subjects began the test at a knee angle of 90 degrees, avoiding any knee valgus movement, and performed a vertical jump by pushing upwards and keeping their legs straight throughout. However, for the countermovement jump subjects began from an upright position, and then made a rapid downward movement to a knee angle of 90°, simultaneously beginning to push-off. Each subject attempted five trials, with 1-min rest intervals. Values for the highest jumps were used in subsequent analyses.

#### Electromyography Recording

Differential bipolar surface electrodes (Bagnoli Desktop EMG systems; Delsys, Inc., Boston, United States) were applied lengthwise over the vastus medialis, vastus lateralis, and rectus femoris muscles of the dominant leg during SJ and CMJ tests, in accordance with European recommendations (Hermens et al., 2000). In order to minimize skin impedance (<5 kΩ) three 4 cm^2^ areas of skin were shaved, abraded and cleaned with an alcohol-ether solution. The vastus medialis electrode was placed at 20% of the distance from the anterior superior iliac spine to the midpoint of the vastus medialis (VM) joint line, the vastus lateralis (VL) electrode was placed at 66% of the distance between the lateral line of the knee joint and the anterior superior iliac spine (Hermens et al., 2000) and the rectus femoris (RF) electrode was located halfway between the greater trochanter and the medial epicondyle of the femur; a reference electrode was attached over the patella of the same leg. The signal was amplified using a differential amplifier (Bagnoli-4 EMG System; Delsys, gain = 1000). EMG signals were filtered to a bandwidth between 20 and 450 Hz to remove unwanted noise and possible movement artifacts in both high- and low-frequency regions. Raw EMG signals were quantified with an analog/digital converter. Before data processing, an additional filter step was carried out with a band pass filter (cut-off frequency = 10 Hz). After that, data were stored in a personal computer for subsequent analysis using the EMG works 4.0.4.3 software (Calculation Toolkit1.5.1.0; Delsys EMG works, Natick, MA, United States). Electromyographic data were expressed as root mean squares (RMS) calculated from the beginning of the takeoff phase until landing. Recordings were made in the concentric phase of the SJ and both the eccentric and the concentric phases of the CMJ. The frequency spectrum was analyzed using a fast Fourier transform approach covering frequencies in the range 5–500 Hz (the signal outside of this range consists mostly of noise) as suggested in a previous investigation (Hammami et al., 2018; Coratella et al., 2020). The window size was 125 ms with a sampling rate of 2000 Hz. The RMS data was normalized relative to the highest EMG activity (% EMG max) according to the formula (Ball and Scurr, 2013):

(taskEMG/referenceEMG)×100

To maximize the consistency of electrode placement from pre-test to post-test, the electrode locations sites were marked and legs were photographed during the first recordings.

#### Isokinetic Testing

To assess quadriceps and hamstring strengths, an isokinetic dynamometer (Biodex, Medical Systems, Inc., Shirley, NY, United States) was used. Subjects were instructed regarding procedures. The warm-up protocol included 10 min of jogging, 5 min of pedaling, and 5 min of stretching of the lower-extremity muscles. Subjects then sat on the dynamometer chair and were immobilized by straps fitted diagonally around the trunk and at the waist, bilateral thighs and shins with the hips and knees flexed to 90° and the center of rotation aligned with the tested knee; they held the sides of the chair with their hands, and were verbally encouraged to maximal effort. Starting with the dominant leg, 5 knee movements were performed back and forth in the concentric mode at low (60°.s^–1^) and medium (120°.s^–1^) angular-velocities. The assessment procedure has been based on previous studies (Coratella et al., 2018). A 2-min period separated each series of movements, and 5 min of rest were allowed before tests on the other leg. A computer program calculated peak torque and average power for the mentioned muscle groups. Gravitational corrections were applied, based on the weight of the limb and accessories. Previous studies have demonstrated a good reliability for repeated isokinetic testing (ICCs of 0.78 and above) (Perrin, 1993).

### Plyometric Training Program

A familiarization session was held 3 days before the intervention, to minimize learning effects. The plyometric program (Table 3) followed a protocol intended to improve performance with minimal risk of injury. The two weekly sessions each comprised three types of combined plyometric exercises: a medium intensity bouncing jump followed by a low to medium intensity hurdle jump and a high intensity drop (Patel, 2014). Training was carried out on a gymnasium floor, using the principle of gradual overload, starting with lower intensity and less complex exercises, and progressing to higher intensity and more complex techniques. Intensity and volume were increased progressively. Thus, the jump height was raised from 0.4 to 0.5 m at the end of the fourth week, and the number of ground contacts was increased every 2 weeks, starting with 72 contacts and reaching 126 contacts. The rest intervals were 48 h between sessions, 120 s between sets and 15 s between repetitions (Ramirez-Campillo et al., 2019). Subjects were encouraged to maximum effort throughout, taking care to avoid knee valgus.

**TABLE 3:** Plyometric training program.

	Weeks												Recovery between sets
	1–2			3–4			5–6			7–8			
Exercise	Reps	Sets	Height	Reps	Sets	Height	Reps	Sets	Height	Reps	Sets	Height	
Bounding jumps	6	4		6	5		6	6		6	7	0.5-m	120 s
Hurdle jumps	6	4	0.4-m	6	5	0.4-m	6	6	0.5-m	6	7		
Drop jumps	6	4		6	5		6	6		6	7		
Total Ground contact per session		72			90			108			126			

*Reps, repetitions; “m”, meter; “s”, second.*

### Statistical Analyses

Statistical analyses were carried out using the SPSS version 20 program for Windows (SPSS, Armonk, NY: IBM Corp). The normality of data was tested using the Shapiro–Wilk test. The Levene test revealed a homogeneity of variance *p* > 5% in all of the tested variables. Descriptive data were presented as adjusted group means and standard deviations. When the assumption of normality was accepted (*p* > 0.05), between-group differences at baseline were examined using independent *t*-tests, and the effect of the intervention was determined by two-way analyses of variance [Experimental vs. Control and Test vs. Retest]. When inter-group baseline differences were found, an analysis of covariance (ANCOVA) was run. Effect sizes were calculated by converting partial eta-squared values to Cohen’s *d*; these were and interpreted according to the Hopkins recommendations: 0.00–0.19: trivial; 0.20–0.59: small: 0.60–1.19: moderate; 1.20–1.99: large; ≥ 2.00: very large (Hopkins et al., 2009). Test–retest reliability was assessed by ICCs; all measures showed an ICC > 0.80 and a coefficient of variation (CV) < 5% (Table 4). When data were not normally distributed, non-parametric tests were used. The Mann–Whitney *U*-statistic, was run to check between-group differences at baseline. The effect of the intervention was set by the Kruskal–Wallis *H*-test and when the significance level reached the threshold, the Wilcoxon signed rank test for paired samples was carried out to assess the group showing significant improvements. The effect size was estimated by the calculation of the epsilon-squared (*E*_*R*_^2^) for the Kruskal–Wallis *H*-test and for the Wilcoxon signed rank test by calculating the correlation coefficients (*r*) as suggested by Tomczak and Ewa (2014). The score for this coefficient ranges between 0 (indicating no relationship) to 1 (indicating a perfect relationship). The alpha level of significance was set at *p* < 0.05 throughout.

**TABLE 4:** Intra-class correlation coefficient showing acceptable reliability for measures of sprint, agility, squat jump, and counter-movement jump test.

	ICC	95%CI	CV
**Sprint times**			
10 m(s)	0.959	0.911 – 0.981	1.80
20 m(s)	0.935	0.858 – 0.970	2.01
30 m(s)	0.994	0.987 – 0.997	1.11
**Change of direction T-test**			
*T*-Test (s)	0.977	0.949 – 0.990	1.25
**Squat Jump**			
Height (cm)	0.997	0.994 – 0.999	1.55
Force (*N*)	0.953	0.898 – 0.979	4.42
Velocity (m.s^–1^)	0.996	0.992 – 0.998	1.14
Power (*W*)	0.990	0.977 – 0.995	4.11
**Counter Movement Jump**			
Height (cm)	0.997	0.994 – 0.999	1.23
Force (*N*)	0.967	0.927 – 0.985	3.70
Velocity (m.s^–1^)	0.998	0.997 – 0.999	0.74
Power (*W*)	0.939	0.865 – 0.972	2.78

*ICC, intra-class correlation coefficient; CI, confidence interval.*

## Results

The PT program did not significantly modify body composition (% body fat, *p* = 0.171, *d* = 0.58). Nevertheless, leg muscle volumes were increased in the experimental group (Δ = 6.26%, *p* = 0.016, *d* = 0.32), as were thigh muscle volumes (Δ = 8.19%, *p* = 0.003, *d* = 0.33), and maximal cross-sectional areas (CSA) (Δ = 3.78%, *p* = 0.002, *d* = 0.61), but paired *t*-tests showed no significant changes in the control group. Further, the group × time interaction revealed that only the maximal CSA of the experimental group showed significant gains [Δ = 9.89%, *p* = 0.028, *d* = 0.95 (moderate)] (Table 5).

**TABLE 5:** Comparison of lower-limb muscle volumes and cross-sectional areas between experimental and control groups before (pre) and after (post) the 8-week trial.

	Experimental group (*n* = 15)			Control group (*n* = 12)			ANCOVA	
	*Pre*	*Post*	*%* Δ	*Pre*	*Post*	*%* Δ	*p*	*D (Cohen)*
*Leg muscle volume (L)*	9.2 ± 1.5	9.7 ± 1.6	6.26 ± 8.53	10.9 ± 2.9	10.9 ± 2.6	–0.3 ± 3	0.087	0.73
*Thigh muscle volume (L)*	6.1 ± 1.3	6.6 ± 1.3	8.19 ± 8.36	8.3 ± 2.6	8.3 ± 2.3	0.7 ± 4.8	0.341	0.40
*Mean thigh CSA (cm^2^)*	155 ± 26	160 ± 21	3.78 ± 10.19	201 ± 64	202 ± 55	1.4 ± 6.8	0.352	0.39
*Maximal thigh CSA (cm^2^)*	226 ± 30	247 ± 34*	9.89 ± 9.40	272 ± 48	270 ± 45	–0.6 ± 3.7	0.028	0.95
*Body fat %*	23.5 ± 4.3	22.0 ± 4.4	–6.3 ± 6.1	27.8 ± 3.6	27.0 ± 3.6	–2.6 ± 7.0	0.171	0.58

ANCOVA, analysis of covariance assessed the statistical significance of intergroup differences.

*p < 0.05.

For sprint and changes of direction tests, although the experimental group tended to improve their running times (10 m Δ = −3.55%, *p* = 0.005, *d* = 0.61; 20 m Δ = −2.02%, *p* = 0.014, *d* = 0.37; 30 m Δ = −2.42%, *p* = 0.003, *d* = 0.42) after the training program, those changes did not reach statistical significance (10 m *p* = 0.154, *d* = 0.41; 20 m *p* = 0.542, *d* = 0.17; 30 m, *p* = 0.454, *d* = 0.21) relative to the control group (Table 6).

**TABLE 6:** Comparison of sprint times between experimental and control groups before (pre) and after (post) the 8-week trial.

	Experimental group (*n* = 15)		Control group (*n* = 12)		Analysis of variance	
	*Pre*	*Post*	*Pre*	*Post*	*p*	*d (Cohen)*
**Sprint times**						
*10 m (s)*	2.15 ± 0.14	2.07 ± 0.10	2.11 ± 0.10	2.12 ± 0.10	0.741^*a*^ 0.266^*b*^ 0.154^*c*^	0.09 0.32 0.41
*20 m (s)*	3.63 ± 0.21	3.56 ± 0.17	3.66 ± 0.19	3.64 ± 0.16	0.273^*a*^ 0.366^*b*^ 0.542^*c*^	0.31 0.26 0.17
*30 m (s)*	5.11 ± 0.30	4.99 ± 0.25	5.16 ± 0.31	5.15 ± 0.26	0.170^*a*^ 0.369^*b*^ 0.454^*c*^	0.39 0.26 0.21
**Ability to change direction**						
*T-test(s)*	11.69 ± 0.59	11.23 ± 0.48*	11.65 ± 0.37	11.87 ± 0.6	0.038^*a*^ 0.395^*b*^ 0.022^*c*^	0.60 0.25 0.67

*ANOVA, a two-way analysis of variance (group × time) assessed the statistical significance of training-related effects.*

“^*a*^” denotes a main effect group; “^*b*^” denotes a main effect of time; “^*c*^” denotes a main effect group × time.

*p < 0.05.

**FIGURE 2:**
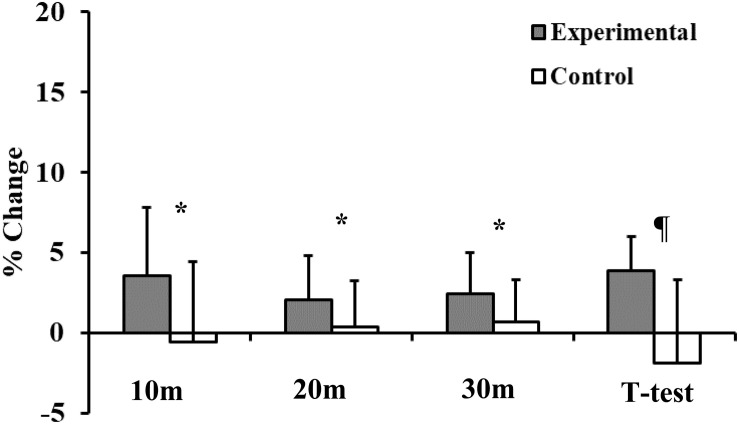
Training associated changes in sprint performance and ability to change direction (*T*-test) in experimental and control groups. *ANOVA: A two-way analysis of variance (group × time) assessed the statistical significance of training-related effects: *p* < 0.05. ¶ANOVA: A two-way analysis of variance (group × time) assessed the statistical significance of training-related effects: *p* < 0.001.

However, the plyometric program significantly decreased the sprinting times of the experimental group during the change of direction *T*-test [Δ = −3.90%; *p* = 0.022; *d* = 0.67 (moderate)] at the end of the training period, whereas times for the control group remained unchanged (*p* = 0.241, *d* = 0.39) (Table 6 and Figure 2).

**FIGURE 3:**
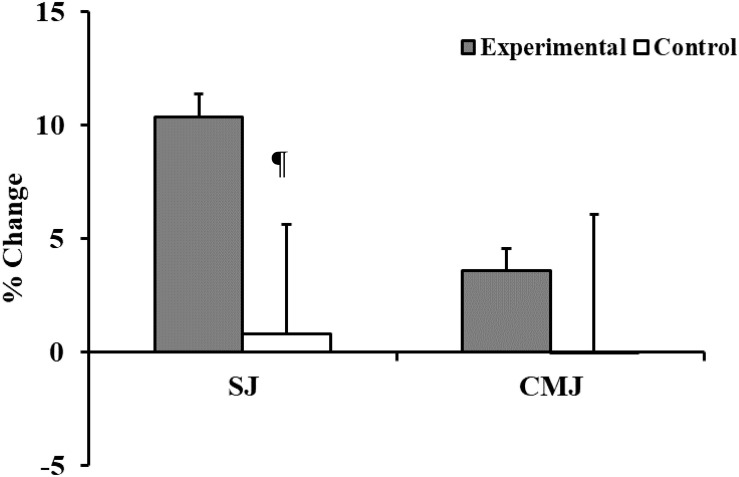
Training associated changes in vertical jump performances (SJ, Squat jump; CMJ, Counter Movement Jump) in experimental and control groups. ¶ANOVA: A two-way analysis of variance (group × time) assessed the statistical significance of training-related effects: *p* < 0.001.

There were notable improvements in squat jump height in the plyometric group (Δ = 10.4%, *p* = 0.001, *d* = 0.67). However, despite a trend to enhancement of the counter-movement jump (Δ = 3.6%), a paired *t*-test revealed its insignificance (*p* = 0.140, *d* = 0.22). Likewise, comparing the experimental to control group, changes did not meet significance (jump height SJ: *p* = 0.198, *d* = 0.37; jump height CMJ: *p* = 0.617, *d* = 0.14) (Table 7 and Figure 3).

**TABLE 7:** Comparison of squat jump and counter movement jumps between experimental and control groups before (pre) and after (post) the 8-week trial.

	Experimental group (*n* = 15)		Control group (*n* = 12)		Analysis of variance	
	*Pre*	*Post*	*Pre*	*Post*	*p*	*d (Cohen)*
SJ height (cm)	33.9 ± 4.6	37.3 ± 4.8	31.3 ± 4.3	31.5 ± 4.3	0.001^*a*^ 0.147^*b*^ 0.198^*c*^	0.97 0.42 0.37
CMJ height (cm)	36.0 ± 4.8	37.2 ± 5.1	33.0 ± 4.1	32.9 ± 4	0.006^*a*^ 0.655^*b*^ 0.617^*c*^	0.82 0.13 0.14

*Analysis of variance a two-way analysis of variance (group × time) assessed the statistical significance of training-related effects.*

*“^*a*^” Denotes a main effect group; “^*b*^” denotes a main effect of time. “^*c*^” denotes a main effect group × time.*

After the intervention period, the Wilcoxon signed rank test revealed that all RMS values measured during the SJ and CMJ were significantly increased in each of the tested muscles of the experimental group (during SJ: RF: *p* = 0.037, *r* = −0.373; VL: *p* = 0.006, *r* = −0.427 and during CMJ: RF: *p* = 0.015, *r* = –0.162; VM: *p* = 0.011, *r* = −0.411; VM: *p* = 0.028, *r* = –0.382) except for the vastus medialis during the SJ. However, the control group, showed no significant changes in these parameter.

**FIGURE 4:**
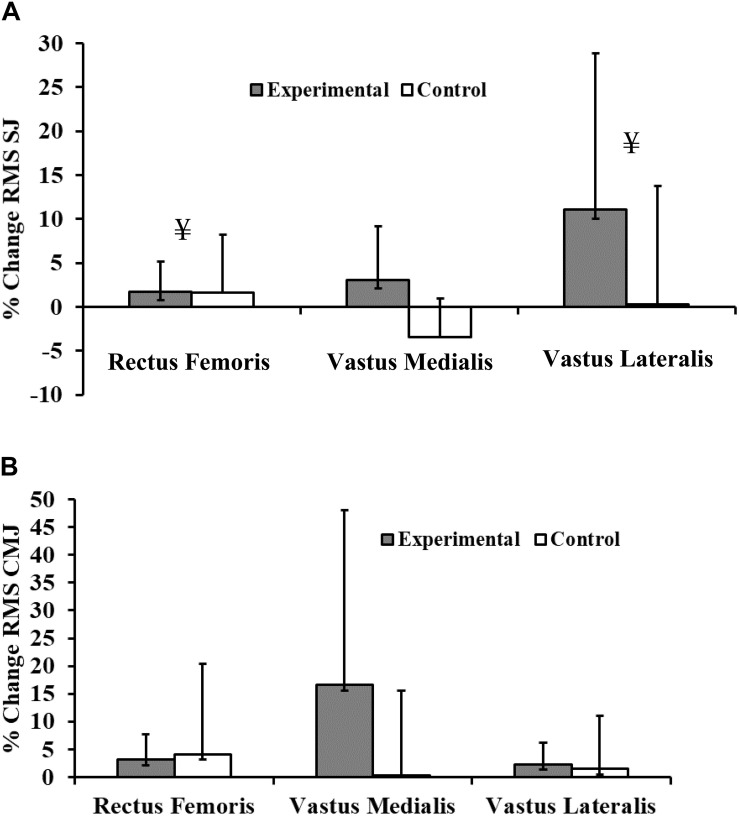
Percentage change of root mean square (RMS) EMG voltage during **(A)** Squat Jump (SJ) and **(B)** Counter-Movement Jump (CMJ) in rectus femoris (RF), vastus medialis (VM), and vastus lateralis (VL) muscles for experimental and control subjects after participation of experimental subjects in the 8-week plyometric training program. ¥ ANOVA: A two-way analysis of variance (group × time) assessed the statistical significance of training-related effects: *p* < 0.001.

Group by time interactions showed significant increases of the RMS values of the experimental group relative to controls only in the rectus femoris and the vastus lateralis (*p* = 0.003, *E*_*R*_^2^ = 0.348; *p* = 0.001, *E*_*R*_^2^ = 0.389) during the squat jump, and any trend to improvement did not reach significance for other RMS measures during the counter-movement jump (Table 8 and Figure 4).

**TABLE 8:** Comparison of root-means-squares of EMG voltages for squat jump and countermovement jump between experimental and control groups before (pre) and after (post) the 8-week trial.

	Experimental group (*n* = 15)		Control group (*n* = 12)		Kruskal–Wallis *H*	
	*Pre*	*Post*	*Pre*	*Post*	*p*	*D (Cohen)*
***Squat jump***						
*RMS.VM (%)*	96.82 ± 4.89	99.53 ± 0.97	99.26 ± 2.17	95.82 ± 5.00	0.088	0.29
*RMS.VL (%)*	91.57 ± 12.10	99.69 ± 0.55*	96.14 ± 8.43	95.47 ± 6.22	0.001	0.39
*RMS.RF (%)*	98.16 ± 3.01	99.81 ± 0.35*	96.46 ± 5.12	97.75 ± 2.35	0.003	0.35
***Countermovement jump***						
*RMS.VM (%)*	86.93 ± 20.66	95.34 ± 13.12	95.97 ± 6.26	95.77 ± 11.86	0.759	0.00
*RMS.VL (%)*	97.12 ± 4.15	99.23 ± 1.31	96.40 ± 5.24	97.51 ± 7.01	0.880	0.00
*RMS.RF (%)*	96.73 ± 4.07	99.60 ± 0.68	95.20 ± 9.84	97.82 ± 7.04	0.578	0.01

Non-parametric Kruskal–Wallis *H*-test assessed a main effect group × time. *p < 0.05.

**FIGURE 5:**
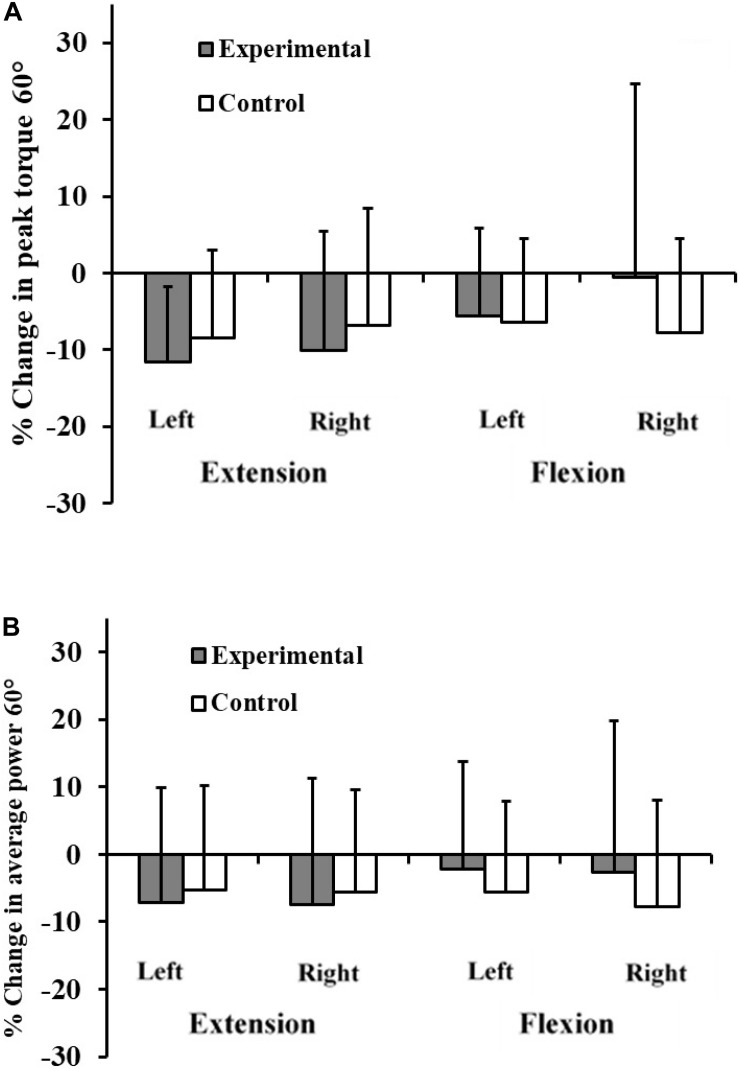
Percentage change in peak torque 60° **(A)** and average power **(B)** for experimental and control subjects after participation of experimental subjects in the 8-week plyometric training program.

**FIGURE 6:**
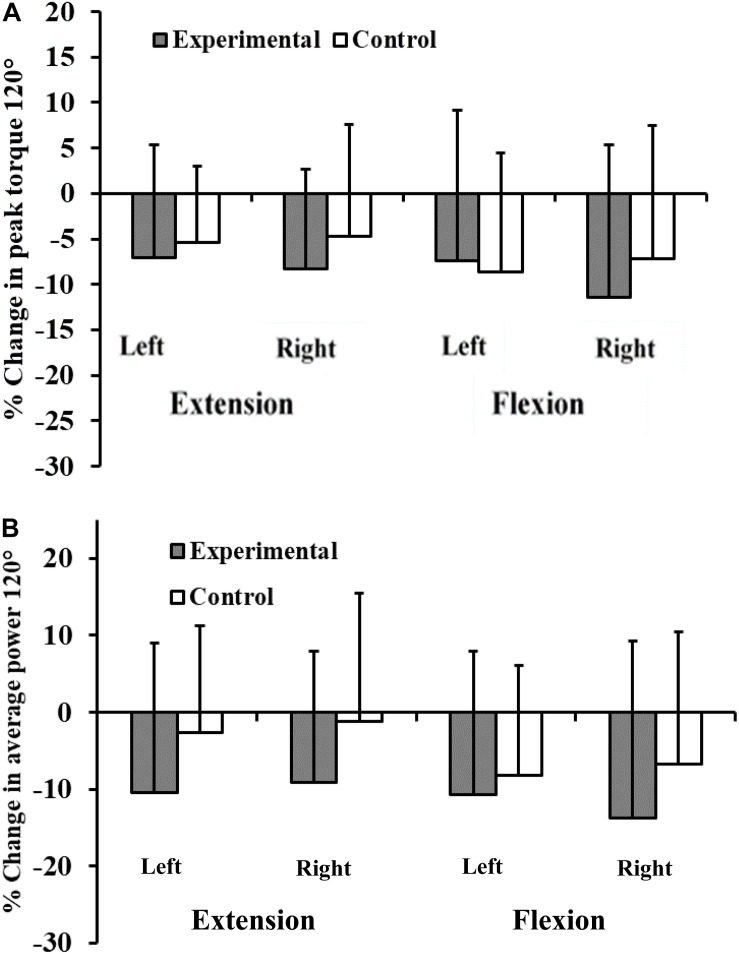
Percentage change in peak torque 120° **(A)** and average power **(B)** for experimental and control subjects after participation of experimental subjects in the 8-week plyometric training program.

Some of the parameters tested also improved in the control group after a corresponding period of their usual training. The group by time interaction showed no significant increases of peak torque, or average power of the experimental group in either leg at the speeds tested (Tables 9, 10 and Figures 5, 6).

**TABLE 9:** Comparison of isokinetic parameters values at 60°.s^–1^ velocity between experimental and control groups before (pre) and after (post) the 8-week trial.

	Experimental group (*n* = 15)		Control group (*n* = 12)		Analysis of variance		ANCOVA	
	*Pre*	*Post*	*Pre*	*Post*	*p*	*d(Cohen)*	*p*	*d (Cohen)*
***Peak torque 60°***								
*Quadriceps right*	116 ± 15	103 ± 17	129 ± 12	118 ± 17			0.380	1.37
*Quadriceps left*	108.4 ± 15.6	96.4 ± 16.7	114 ± 20	105 ± 17			0.375	0.37
*Hamstrings right*	75.7 ± 10.9	71.3 ± 12.5	78.3 ± 7.7	73.0 ± 9.4	0.459^*a*^ 0.096^*b*^ 0.887^*c*^	0.21 0.48 0.00		
*Hamstrings left*	70.5 ± 12.4	68.1 ± 10.7	78.2 ± 10.7	72.1 ± 13.5	0.077^*a*^ 0.199^*b*^ 0.578^*c*^	0.51 0.37 0.16		
***Average Power 60°***								
*Quadriceps right*	85.3 ± 12.0	80.3 ± 21.5	98.4 ± 13.3	92.9 ± 18.0			0.633	0.20
*Quadriceps left*	80.3 ± 9.9	74.3 ± 17.3	91.2 ± 21.9	85.1 ± 19.1	0.026^*a*^ 0.208^*b*^ 0.996^*c*^	0.65 0.36 0.00		
*Hamstrings right*	67.9 ± 10.5	65.9 ± 13.2	70.3 ± 11.1	65.6 ± 9.3	0.729^*a*^ 0.284^*b*^ 0.669^*c*^	0.09 0.31 0.13		
*Hamstrings left*	64.2 ± 9.9	61.4 ± 12.1	72.3 ± 14.9	66.4 ± 15.8	0.073^*a*^ 0.232^*b*^ 0.665^*c*^	0.52 0.34 0.13		

*ANOVA, a two-way analysis of variance (group × time) assessed the statistical significance of training-related effects.*

“^*a*^” Denotes a main effect group; “^*b*^” denotes a main effect of time. “^*c*^” denotes a main effect group × time.

*p < 0.05.

**TABLE 10:** Comparison of isokinetic parameters values at 120°.s^–1^ velocity between experimental and control groups before (pre) and after (post) the 8-week trial.

	Experimental group (*n* = 15)		Control group (*n* = 12)		Analysis of variance	
	*Pre*	*Post*	*Pre*	*Post*	*p*	*D (Cohen)*
***Peak torque 120°***						
*Quadriceps right*	84.6 ± 13.6	78.4 ± 15.5	87.6 ± 9.5	82.7 ± 9.9	0.295^*a*^ 0.116^*b*^ 0.862^*c*^	0.30 0.45 0.06
*Quadriceps left*	80.5 ± 11.6	73.8 ± 14.1	82.1 ± 11.1	77.2 ± 7.5	0.435^*a*^ 0.075^*b*^ 0.781^*c*^	0.22 0.51 0.09
*Hamstrings right*	63.1 ± 12.2	57.7 ± 12.9	61.9 ± 7.4	55.8 ± 4.4	0.586^*a*^ 0.047^*b*^ 0.898^*c*^	0.16 0.58 0.00
*Hamstrings left*	61.1 ± 11.9	53.6 ± 13.1	63.2 ± 9.1	57.9 ± 7.9	0.289^*a*^ 0.038^*b*^ 0.721^*c*^	0.30 0.61 0.11
***Average Power 120°***						
*Quadriceps right*	122 ± 25	109 ± 29	128 ± 15	123.4 ± 16.7	0.125^*a*^ 0.161^*b*^ 0.467^*c*^	0.44 0.40 0.21
*Quadriceps left*	114 ± 20	103 ± 27	120 ± 23	115.8 ± 15.7	0.130^*a*^ 0.248^*b*^ 0.610^*c*^	0.43 0.33 0.14
*Hamstrings right*	102 ± 24	89 ± 24	99 ± 13	89.9 ± 10	0.847^*a*^ 0.048^*b*^ 0.777^*c*^	0.06 0.57 0.09
*Hamstrings left*	97 ± 19	129 ± 184	102 ± 21	92.6 ± 15.3	0.559^*a*^ 0.662^*b*^ 0.440^*c*^	0,17 0.13 0.22

*ANOVA, a two-way analysis of variance (group × time) assessed the statistical significance of training-related effects.*

*“^*a*^” Denotes a main effect group; “^*b*^” denotes a main effect of time. “^*c*^” denotes a main effect group × time.*

## Discussion

The 8-week in-season plyometric program, was not sufficient to induce significant improvements of physical performance. Although the paired *t*-test showed trends of enhancements in sprinting speed, squat and counter-movement jumping, peak torque and average leg power performances in the experimental relative to the control group, group × time interactions did not reach significance. However, there were significant gains in the ability to change direction (*d* = 0.67), in the RMS values for the vastus medialis muscles (*d* = 0.59) and in the maximal cross-sectional muscle area (*d* = 0.95).

### Anthropometrics

A significant gain in maximal thigh CSA with trends to increases of thigh and leg muscles volumes were seen at the end of the PT program. In agreement with our results, Chelly et al. (2015) demonstrated that following a 10-week plyometric program, total leg and thigh muscle volumes remained unchanged, whereas the CSA of the thigh was significantly increased. As well, Potteiger et al. (1999) showed a significant increase of the CSA after an 8-week PT in physical active men. Miller et al. (1993) had earlier demonstrated a significant correlation between strength and muscle CSA. Likewise, Gabriel et al. (2006) argued that strength and power adaptations were largely associated with increases in muscle CSA. Similarly, the significant enhancement of CSA seen in this study could be a major contributor to the enhanced jumps, sprints and change of direction performances.

### Sprint Performance

Trends to faster sprinting of the experimental group over distances of 10, 20, and 30 m did not reach the significance threshold relative to the control group, a finding comparable with some previous trials. Thus, Bouteraa et al. (2018) saw no improvements of sprinting in female U17 basketball players, after 8 weeks of a biweekly in-season combined balance and PT program. Similarly, Markovic et al. (2007) observed no significant improvement in the 20 m sprint speeds of male physical education students after a 10-week plyometric program. PT reduces ground contact time, which might be expected to shorten sprint times (Rimmer and Sleivert, 2000); the lack of significant change in our study could reflect in part the contact times for the plyometric exercises, which were longer than the contact times during sprints. Arazi et al. (2014) observed a 15.9% decrease in the 20-m sprint times of initially untrained women after 6 weeks of PT. Ramirez-Campillo et al. (2018b) also saw significant enhancement in sprint performance in female soccer players after 8 weeks of PT. As well, in a more recent study Bianchi et al. (2019) showed similar improvement in 10 and 30 m sprint performance in youth football players after a 6-week of PT. Discrepancies could be due to differences in age, program period and duration. In addition, it was previously demonstrated that after PT, beginners tended to show greater increases in sprint performance than experienced players (Delecluse, 1997). Gains are likely due to activation of the stretch shortening cycle, increasing the ability of the muscle tendon unit to produce maximal force rapidly (Sáez de Villarreal et al., 2012). Possibly, the adoption of a training program that incorporated more horizontal acceleration (e.g., bounding and form running) might have had a greater effect upon sprint times (Sáez de Villarreal et al., 2012).

### Change of Direction

The experimental group markedly increased their ability to change direction rapidly. This could reflect neural adaptations such as improved muscle activation strategies (better inter- and intra-muscular coordination) and an increased neural drive to agonist muscles, allowing players to switch rapidly between deceleration and acceleration (Markovic and Mikulic, 2010). Bouteraa et al. (2018) also demonstrated enhanced scores on the modified Illinois change of direction test in female adolescent basketball players after 8 weeks of combined plyometric and balance training program, although Meszler and Váczi (2019) showed that the 7-week in-season PT was not effective in agility development of U17 female basketball players. Likewise, Lehnert et al. (2013) found no significant changes in this characteristic of elite basketball players after 6 weeks of PT. Discrepancies in results could be due to methodological differences in training programs (duration, intensity, number of contact, period of the season…) as well as the subjects’ characteristics (gender, age, years of practice…).

### Jumping Tests

The present program did not enhance the jumping ability of the experimental group significantly relative to controls. Bouteraa et al. (2018) also found no significant effects of a plyometric regimen on either squat- or counter-movement jumps, but in contrast to our findings, Attene et al. (2015) observed a 15.4% gain of standing jump and an 11.3% increase in counter-movement jump performance, in 15-year-old female basketball players after 6 weeks of PT. These divergent results may be due to the specificities of the selected sample populations (age, experience with plyometric drills), the exercises chosen and the nature of the contractions they involve. The trends to an improvement of the SJ (10.4 %) and CMJ (3.6%) in our study run counter to the argument of Markovic et al. (2007) and Stojanovic et al. (2017) that PT produces somewhat greater positive effects in the slow SSC jumps of the CMJ (8.7%) than in concentric-only jumps like SJ (4.7%). Any enhancements could be due to both structural (enhanced elastic properties of the musculo-tendinous unit) and neural factors. The lack of significant results in the present study, could reflect firstly training status and experience of the athletes (Stojanovic et al., 2017) and secondly to the fact that although our basketball players had not previously been subjected to extended PT, they had carried out a large number of jumps during practice and games (Ziv and Lidor, 2010). Furthermore, it could be that the speed of execution as well as the angle adopted during plyometric exercise was insufficient to induce an improvement in muscle stiffness and thus greater storage and release of elastic energy (Sale and MacDougall, 1981). Moreover, Stojanovic et al. (2017) argued that gains in the counter-movement jump height were unlikely with less than 10 weeks of PT. At any event, the number of jumps performed during the present training program seems to have been insufficient to induce significant improvements.

### Electromyography

The extreme force and tension demanded by a plyometric regimen leads to neurophysiological adaptations (Ebben et al., 2008). Our results demonstrated a significant training-induced rise of RMS values for the rectus femoris and the vastus lateralis during squat jumping. Moreover, data for the other tested muscles showed parallel but not significant trends.

Bosco et al. (1982) have shown that quadriceps activation contributes 50% of the total work in a vertical jump during SJ and CMJ, which goes in tandem with the trends to an increase of the SJ height (10.4 %) and the significant enhancement of RMS of the rectus femoris and the vastus lateralis that we observed. Hammami et al. (2019) also noted that an 8-week plyometric program (two times a week) based on hurdle and drop jumps significantly improved the RMS values for the rectus femoris in elite male soccer players (age = 15.7 ± 0.2 years) during squat jumping, although they also found significant increases in vastus medialis RMS values during the squat jump and for both muscles during the counter-movement jump. Similarly, Toumi et al. (2004) found significant improvement of RMS values for the knee extensor muscles (vastus medialis and vastus lateralis) during SJ after 8 weeks of PT. In contrast to the current findings, Toumi et al. (2004) also found an improvement in RMS values during the CMJ. On the other hand, Mehdipour et al. (2008) saw no effect on RMS for the rectoris femoris and right thigh muscles after 6 weeks of tri-weekly PT. These disparate results may reflect differences in the type and intensity of programs and the height of the jumps undertaken. Thus Ebben et al. (2008) argued that a 15–24-cm cone hop, a 61-cm box jump, and tuck jumps resulted in greater increases of integrated quadriceps RMS than exercises such as the single-leg jump, and depth jumps from 30- and 61-cm boxes. Some studies may also have used drills that failed to elicit appropriate actions from muscles fibers and motor units (Ebben et al., 2008). In this respect, Häkkinen (1994) stated that a major part of the improvements during the initial weeks in ballistic-type strength training is probably due to adaptations of the neural system, such as increased motor unit firing frequency, improved motor unit synchronization, increased motor unit excitability, an increase in efferent motor drive, and improved co-activation of the synergist muscles.

The EMG signal is sensitive to the layer of fatty tissue lying between the electrode and the muscle, and this can lead to inaccuracy in the results obtained with the application of surface electromyography (Farina and Mesin, 2005; Bartuzi et al., 2010). Increases in sub-cutaneous fat could weaken the EMG signal (Kuiken et al., 2003). However, we ensured that the percentage of body fat did not change significantly during training (Table 5). Further, it seems that the exercise intensity (number of jumps per session and hurdle jump height) was insufficient to induce more significant improvements in both neural and physical performance.

### Power Assessment

The current training program did not augment average power or peak torque. Likewise, Meszler and Váczi (2019) did not observe any significant changes of the maximal voluntary flexor or extensor isometric torque at 60°/s velocity in U-17 female basketball players. Similarly, Wilkerson et al. (2004) found no significant group × time interaction for peak quadriceps torque after female collegiate basketball players (age = 19.6 ± 1.4 years) had undertaken a 6-week plyometric pre-season program, although they did see gains in the hamstrings peak torque. Fry et al. (1992) suggested that generalized strength training may not improve the hamstrings peak torque, unless isolated hamstring exercises are incorporated into the protocol. However, Hewett et al. (1996) tested the effect of a 6 weeks plyometric and strength program on female volleyball players (age = 15.0 ± 0.6 years) and contrary to the present study, they found significant gains in average power [dominant leg (*p* < 0.001), non-dominant leg (*p* < 0.05), and the peak torque of the non-dominant leg (*p* < 0.01)]. Myer et al. (2006) also demonstrated that 7 weeks of PT (three times a week) at a velocity of 300°.s^–1^ significantly increased the peak isokinetic torque of the hamstrings (*p* < 0.01) in female athletes (age = 15.9 ± 0.8 years).

Gabriel et al. (2006) suggested that strength or power adaptations were largely associated with increases in the root-cross-sectional area of the muscle. However, the current results demonstrated a significant increase in the maximal CSA of leg muscle without gains in peak torque or average power. It would appear that the increase in muscle power is dependent on the improvement of mean thigh CSA (which was not significantly increased in this study) and not on maximal thigh CSA. In this context Moritani et al. (1992) reported that during the first 6–8 weeks of strength and power training, neural adjustment and adaptation, increases in motor unit recruitment, and synchronization of motor unit firing are the dominant factors in gains of performance, rather than muscle hypertrophy. Furthermore, our results are going against what has been suggested by Bobbert et al. (1987) that 20 and 40 cm height jumps provide higher peak moment and power output about ankles and lower joint reaction forces then the initial height of 60 cm. Given that within the current program the heights of 40 and 50 cm have not led to important improvements, it is possible that for players who are already accustomed to performing many jumps during their usually activities, it is necessary to extend the intensity of the jumps and to plan programs with higher heights. The neuromuscular adaptations also seem responsible for the improvements of sprinting, agility and squat jumping (height and speed).

## Limitations

An extension of the duration of the plyometric program and an increase in exercise intensity might have enabled participants to achieve significant improvements in sprints and jumping performance. However, since vertical jumping and muscle strength are crucial qualities for basketball players, the absence of increases in strength points to a possible need to combine resistance training with a plyometric regimen in order to maximize gains in basketball playing ability. The current investigation was conducted on adult female basketball players at a specific level of competition, and there is a need to extend these results to cover other age and gender groups, and other skill levels. It also remains interesting to see and compare the observed trends to enhancements (even if they are not all statistically significant).

## Conclusion

The present study underlines the limited practical contribution of the integration of an 8-week plyometric program into standard in-season skill-based training in terms of improvements in sprinting, jumping and the ability to change direction in elite female basketball players. Given that some previous studies of adult basketball players have yielded a more positive response, we may suggest that the pattern of PT chosen (72–126 jumps per session) was insufficient to enhance determinants of basketball performance, despite some trends suggestive of neural adaptations.

## Data Availability Statement

The original contributions presented in the study are included in the article/supplementary material, further inquiries can be directed to the corresponding author/s.

## Ethics Statement

The studies involving human participants were reviewed and approved by the ISSEP – Ksar Said Institutional Review Committee for the ethical use of human subjects, according to current national laws and regulations. Written informed consent to participate in this study was provided by the participants’ legal guardian/next of kin.

## Author Contributions

MSC and MJ contributed to formal analysis and supervised the study. YC, MH, and GA investigated the study and performed the methodology. MSC and YC contributed to the project administration. YC, MH, and MSC wrote the original draft of the manuscript. RS, MSC, and KS wrote, reviewed, and edited the manuscript. All the authors contributed to the article and approved the submitted version.

## Conflict of Interest

The authors declare that the research was conducted in the absence of any commercial or financial relationships that could be construed as a potential conflict of interest.
